# Nanoscience‐Based Strategies to Engineer Antimicrobial Surfaces

**DOI:** 10.1002/advs.201700892

**Published:** 2018-03-08

**Authors:** Serena Rigo, Chao Cai, Gesine Gunkel‐Grabole, Lionel Maurizi, Xiaoyan Zhang, Jian Xu, Cornelia G. Palivan

**Affiliations:** ^1^ Chemistry Department University of Basel Mattenstrasse 24a 4058 Basel Switzerland; ^2^ Beijing National Laboratory for Molecular Sciences Laboratory of Polymer Physics and Chemistry Institute of Chemistry Chinese Academy of Sciences Zhongguangcun North First Street 2 100190 Beijing P. R. China

**Keywords:** antimicrobial surfaces, biofilms, nanoscience, smart surfaces

## Abstract

Microbial contamination and biofilm formation of medical devices is a major issue associated with medical complications and increased costs. Consequently, there is a growing need for novel strategies and exploitation of nanoscience‐based technologies to reduce the interaction of bacteria and microbes with synthetic surfaces. This article focuses on surfaces that are nanostructured, have functional coatings, and generate or release antimicrobial compounds, including “smart surfaces” producing antibiotics on demand. Key requirements for successful antimicrobial surfaces including biocompatibility, mechanical stability, durability, and efficiency are discussed and illustrated with examples of the recent literature. Various nanoscience‐based technologies are described along with new concepts, their advantages, and remaining open questions. Although at an early stage of research, nanoscience‐based strategies for creating antimicrobial surfaces have the advantage of acting at the molecular level, potentially making them more efficient under specific conditions. Moreover, the interface can be fine tuned and specific interactions that depend on the location of the device can be addressed. Finally, remaining important challenges are identified: improvement of the efficacy for long‐term use, extension of the application range to a large spectrum of bacteria, standardized evaluation assays, and combination of passive and active approaches in a single surface to produce multifunctional surfaces.

## 1 Introduction

Surfaces in contact with biological fluids are prone to colonization with bacteria, which severely hamper their function and can cause infections and other unwanted side effects. Several types of medical implants and devices (i.e., catheters, tubes, artificial joints, etc.) are used in various parts of the body to replace the function of missing or disabled organs/joints or to facilitate tissue repair. For example, 17.5% of patients in European hospitals have a urinary catheter,^1^ and catheter‐associated urinary tract infections, often caused by *Escherichia coli*,^2^ are a common cause of secondary blood stream infections.^1^ Moreover, with the growth of an elderly population there is an increasing need for medical implant devices such as feeding‐ or tracheostomy tubes.^3^ Continued function of these implanted devices is a central aspect to improve the quality of life of patients; therefore, complications such as device‐associated infections (DAIs) are one of the most important challenges in this field. Bacteria can cause DAI if they colonize the device surface and grow into biofilms that induce a dynamic and multifaceted process, in which products like signaling molecules are actively shared and exchanged. The different states of biofilm formation include the transition of planktonic to sessile bacteria, attachment and cell‐to‐cell adhesion, growth and maturation, and detachment and dissolution to spread and colonize new areas. The microorganisms within a biofilm are embedded and protected by self‐produced extracellular polymeric substances (EPSs), which contain polysaccharides, proteins, extracellular DNA, glycoproteins, and other natural polymers. Treating DAI is difficult, because the bacteria in a biofilm are not easily accessible, the efficacy of antibiotic treatments is low due to their resistance to antibiotics^4^ and further reduced since concentration of the antibiotic below the minimal inhibitory concentration (MIC) even supports biofilm formation,^5^ thereby inducing ineffective treatment against nonmultidrug‐resistant bacteria strains, which tend to form more robust biofilms.^6^ Moreover, DAIs of orthopedic devices, which typically have low infection rates,^7^ are particularly difficult to treat. The device must be removed and replaced after disinfection of the infected area. Furthermore, replacement due to an infection is several times more costly than the primary implantation or replacement of a noninfected implant.^8^

These examples illustrate the importance and need for the development of new methods which do not only rely on conventional administration of antibiotics to prevent bacterial attachment and thus DAI. Nanoscience‐based solutions are of particular interest, because they can prolong the utility of antimicrobial surfaces by providing specific surface modifications and allow attachment/insertion of active compounds to achieve specific antimicrobial properties. Effective antimicrobial surface coatings are generally based on i) antiadhesive properties that prevent adherence of bacteria or ii) bactericidal strategies that kill organisms either before or after contact with the surface. The designs applied in nanoscience‐based antimicrobial surfaces implement passive strategies (antiadhesive properties), active strategies (bactericidal activity), or in more recent examples combine both of them to gain in overall efficacy. A large variety of surfaces are obtained, depending on the modification of the solid support (physical, chemical, or both) and the supplementary association with antibacterial components (bio‐, synthetic molecules, membranes, and assemblies). Antibacterial surfaces based on nanoscience approaches include: i) hydrogels or hydrogel‐like films with temporal release of active agents,^9^ ii) solid supports decorated with: polymer brushes,^10^ nanoparticles,^10^, ^11^ nanocarriers,^12^ nanostructures,^13^ and nanoreactors, which are catalytically active nanocompartments, which produce reactive agents in situ,^14^ and iii) micro‐ and nanopatterned surface structures.^15^ Nanoscience‐based strategies to design antimicrobial surfaces have the advantage of being bottom‐up approaches, which allow combination of different biosynthetic, and synthetic compounds and assemblies at molecular level. Thereby, better local control of the resulting properties and functionality is achieved compared to other types of surfaces used in implants. In addition, both passive strategies based on nano‐ and microstructuring of the surface, and active strategies involving the release of antimicrobial compounds allow fine tuning of the components, such to improve the local efficacy with minimal side effects (especially in the case of administration of antibiotics).

**Figure 1:**
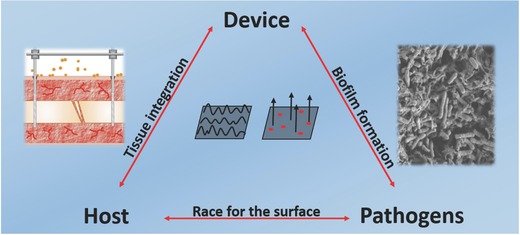
Interactions between device,^17^ host, and pathogens^18^ are the deciding factors for successful insertion of the device during implantation. Tissue integration should be promoted while preventing biofilm formation. Possible nanosolutions in topography and releasing active agents will be discussed in this review. Reproduced with permission.^17^ Copyright 2013, Elsevier.

Successful implantation and implant survival depends on interactions between the device, the host, and the bacteria (**Figure** **1**). They influence the ease with which an implant integrates into the biological environment of the host's body and their ability to prevent bacterial growth. To this end, there are various parameters that need to be considered; i) the surface and material properties of the device, ii) the type of pathogen, and iii) the strength of the host's immune system, which is decreased around synthetic material due to frustrated phagocytosis which are not able to efficiently kill bacteria close to the implant, a nonphagocytosable surface.^16^ These interactions are critical factors in choosing the appropriate surface for a device used in specific treatment. Competition between bacteria and cells for the implant surface is crucial, and the risk of DAI is significantly decreased as soon as the implant is completely colonized by the host's cells, since there is no free surface left for attachment and proliferation of bacteria.

In this review, we focus on innovative and promising ideas, and recent developments in nanoscience‐based strategies to engineer antimicrobial surfaces for biomedical devices and on the key features needed for a successful antimicrobial surface, which are mechanical stability, biocompatibility, antimicrobial efficiency, antimicrobial durability, and avoid bacterial resistance. For details on biofilm formation,^19^ antimicrobial peptide (AMP) activity,^20^ and drug delivery,[[qv: 20a,21]] the reader is referred to excellent reviews by others. However, the domain of development of antimicrobial surfaces is still controversial due to the biocomplexity of the medical conditions and a lack of standard methods to evaluate the biologic reply of such surfaces. The physicochemical properties of such surfaces are characterized normally to evaluate their wettability, coating thickness, or specific surface properties. A plethora of established methods is available for characterization of these parameters, including microscopy techniques (electron microscopy, atomic force microscopy) and methods to investigate specific surface interactions (e.g., surface plasmon resonance) or the chemical composition (e.g., X‐ray photoelectron spectroscopy). Evaluation of the antibacterial and antimicrobial properties, however, is more complex and lacks comparability.

Various bioassays are used to evaluate the efficacy of the antibacterial surfaces in vitro, ranging from basic tests (agar diffusion,[[qv: 14a]] minimum inhibitory concentration,^22^ and biofilm inhibitory concentration^6^) up to more elaborated antimicrobial activity assays (biofilm formation, colony‐forming unit counting,^23^ enzymatic activity, polymerase chain reaction, etc.). However, problems not solved yet include, but are not limited to: i) the use of single or multiple strains, ii) the selection of the relevant strains for a specific application or for a general use of such surfaces, iii) the conditions that are biorelevant for a specific application (e.g., media, buffers, and incubation times), and iv) the selection of ex vivo testing methodology. This complex scenario of requirements combined with the large diversity of bioassays and conditions make extremely difficult the comparison of the data from different laboratories, and to achieve a clear overview of the key factors producing a desired bioreply for such antimicrobial surfaces.

In this respect, this article serves to indicate the efforts related to specific steps necessary to achieve a successful antimicrobial surface; therefore, we concentrate on the first step, particularly the design of antimicrobial surfaces by nanoscience‐based approaches. We focused our review on examples that support this first step of surface modification and will indicate the advantages and limitations remaining to be solved.

Nanoscience‐based surface modifications are still at an early stage of research in the context of antibacterial surfaces, and they offer solutions that combine chemistry, physics, and materials science to fight bacterial growth at the molecular and local level. Surface‐related examples of strategies that have the potential to protect medical devices against bacteria attachment and biofilm formation are presented and critically analyzed. However, interdisciplinary efforts and combined expertize are essential to advance the development of efficient antimicrobial surfaces by determining the relations between molecular properties of such surfaces at nano‐ and micoscale and their biofunctionality.

## 2 Key Features for the Design of Efficient Antimicrobial Surfaces

**Figure 2:**
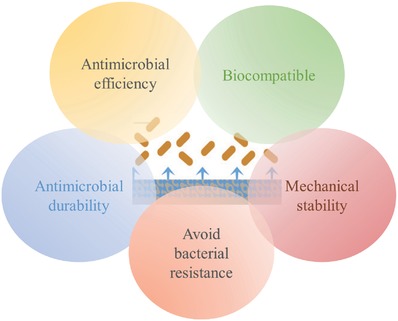
Requirements of antimicrobial and antibacterial surfaces for preventing DAI.

In order to prevent biofilm formation on implanted devices, various substances and technological approaches have been proposed and tested that fulfill the specific constraints related to the production of devices with antibacterial surfaces. These requirements include easy and reproducible production,[[qv: 9a]] adequate sterilization,^24^ and possible repair without increasing the damage.^25^ Moreover, the several requirements have been identified that are essential for an efficient antimicrobial surface (**Figure** **2**), including specific properties and functionalities, which can change depending on the needed function and location of the aimed surface. In the following, current developments and importance of each of these parameters are highlighted.

### 2.1 Mechanical Stability

Antimicrobial coatings need to withstand the mechanical stresses involved in surgical implant insertion and their use in vivo while maintaining long‐term stability^26^ and specific mechanical and physical properties (elasticity, yield stress, ductility, time‐dependent deformation, ultimate strength, fatigue strength, and hardness). For example, the mechanical stability of covalently attached GL13K, a cationic antimicrobial peptide, has been shown to be unaffected by ultrasonication which simulates in vivo fluid flow forces.^27^ Coatings need to be resistant toward any kind of degradation, as well as have mechanical, and thermochemical stability for the long‐term retention of the coated substance and thereby ensuring the antimicrobial effectiveness.^27^, ^28^ Polytetrafluoroethylene coating on orthodontic brackets minimized biofilm formation, but it was partially abraded on surfaces exposed to high shear forces.^29^ These examples underline that the destructive factors are different for each specific environment; thus, the implant surface needs to be designed specifically for the desired application in order to achieve required mechanical stability and minimize the amount of wear particles as much as possible.^30^

### 2.2 Biocompatibility

The compatibility with tissue, biological fluids, or a living system of a surface coating is crucial and the material should not be toxic, injurious, or physiologically reactive or cause unwanted immune responses. Testing the biocompatibility of coatings is generally performed in vitro by evaluating the interactions between the coatings and recognized cell culture lines.^31^ For example, no cytotoxicity was observed in mammalian cells for the release of AMPs when covalently attached,^27^ embedded in polysaccharide films,^32^ or physically adsorbed on titanium‐oxide nanotubes.[[qv: 31b]] However, such in vitro conditions do not adequately address the acceptability of these AMP‐modified materials in environments in contact with blood for a prolonged time (e.g., central venous catheters), and interactions of these coatings with blood are critical for the functioning of the device. The hemocompatibility of coatings has been studied by determining platelet adhesion and thrombin generation in human blood,[[qv: 31b]] even under high pressure and high‐shear arterial flow.^33^ Furthermore, the surfaces of long‐term implants need to allow cells to adhere to the implant surface while suppressing the attachment of bacteria. Precoating of meshes with human cells has shown to be promising to reduce inflammation^34^ in tissue repair. Titanium widely used for long‐term implants is often coated or surface treated^35^ to enhance implant survival. Studies to test viability and metabolism of host cells separately with the capability of bacteria to form biofilms on implant surfaces represent a good first step for predicting that will be able to first colonize the implant surface. To this end, silver‐releasing hydroxyapatite coatings with fibronectin on titanium alloy (Ti‐6Al‐4 V) surfaces have shown a high bacteria killing property (*Staphylococcus aureus)* while being nontoxic to host cells (fibroblasts).^36^ However, biofilms were able to grow on surfaces of titanium, titanium–zirconium alloy, and zirconium oxides with comparable roughness/smoothness,^37^ and although the roughness or hydrophobicity did not have a decisive influence, the lowest biofilm formation was observed on the roughest titanium surface. Nevertheless, coculture studies are needed in order to reliably predict the chances of host cells winning the race for the surface. Such studies with human gingival fibroblasts (HGFs) revealed that bacteria decreased the amount of HGF cells on all but the smooth titanium surface, which supported the best soft tissue integration.^37^ However, the exact influence of the roughness, as a key physical molecular aspect of the surface is still unclear[[qv: 15c]] and discussed controversially in literature, other works observed no significant differences between rough and smooth surfaces for implant survival,^38^ and even significantly higher survival rates for rough surfaces.^39^ In a comparison to untreated titanium (Un‐Ti), surfaces with sulphuric acid treated titanium surfaces and sulphuric acid treated titanium surfaces with immobilized chitosan (SA‐CS‐Ti) in coculture studies, SA‐CS‐Ti surfaces showed the lowest bacteria adhesion both after 30 min and 4 h (when *S. aureus* was incubated together with osteoblasts). However, from 30 min to 4 h the amount of osteoblasts only increased on Un‐Ti surfaces. This could be a consequence of the sulphuric acid treatment increasing the roughness and enhancing the attachment of cells and bacteria, while chitosan only minimized bacterial attachment.^40^ Such coculture studies^41^ provide important data for predicting the chances of successful osseo‐ or soft tissue integration. Combination of surface treatment (control of the physicochemical properties) and release of bactericidal agents seems to be the most promising strategy for prevent DAI, although studies over longer periods of time are still needed.

### 2.3 Antimicrobial Efficiency

Antimicrobial efficiency refers to an evaluation of the ability of the active substrate to decrease bacteria population over time. Most in vitro antimicrobial tests use a static “closed” testing system,[[qv: 9b,34b,42]] whereas in vivo the implant has to face a dynamic, continuously changing, mechanically unstable, and predominantly fluid environment.[[qv: 9b,12,34a,43]] To date, there is no widely accepted methodology available to precisely and reproducibly evaluate the antimicrobial efficiency of new nano‐based solutions proposed for antimicrobial surfaces.^44^ In this respect, controlled and standardized testing conditions that closely mimic the human in vivo environment need to be developed for the evaluation of antimicrobial efficiency. In addition, the specificity of antimicrobial surfaces for certain bacterial species and/or strains may preclude their use as broad therapeutic strategies.

### 2.4 Antimicrobial Durability

Antimicrobial durability ensures that the surface maintains its function over a lengthy time period under defined conditions without excessive expenditure on maintenance or repair. The implementation of durability of the antimicrobial effect is related to the mode of action, mainly “contact killing” or antimicrobial agent‐eluting mode. While in the case of drug delivery systems the release of antimicrobial compounds is mainly relevant up to 24 h (retard release), for antibiotic‐eluting coatings, the finite release property (i.e., after a certain time point they will release below minimal inhibitory concentration of the antibiotic) limits their use in implants.^45^ For short‐term implants (e.g., catheters), the finite release property is not a problem as the implant is removed before full release of the antibiotic.^46^ However, for long‐term implants, continuous strong release of antibiotics is crucial within the first few hours postimplantation, while the immune system is weakened and the implant is most susceptible to bacterial colonization. Antimicrobial surfaces based on degradable polymers[[qv: 11b]] and multilayered surfaces[[qv: 31b]] show promise for achieving long‐term antimicrobial durability, because their degradation can be designed to control the rate and quantity of antimicrobial release for more efficient effects. In this respect, it is essential to exploit the potential of nanostructured surfaces to obtain long‐term antimicrobial activities.^47^ For instance, nanoengineered topography can highly reduce bacteria attachment by decreasing the area of contact between surface and microbes,^48^ and the attachment of blood cells to surfaces can be reduced by coating them with tethered perfluorocarbon chains to avoid thrombosis.^33^ Therefore, since the antimicrobial effect is affected by a combination of surface topography and chemical modification, these have to be studied systematically to optimize their antimicrobial efficiency.

In addition, different strategies for regeneration of antimicrobial activity have been developed. For example, a regenerative silver–zwitterion organic–inorganic antimicrobial nanocomposite by loading zwitterionic polymer brushes with Ag^+^ ions was described, and this was able to produce successive in situ reduction to Ag nanoparticles by UV irradiation.^10^ An alternative solution is to use “smart antimicrobial surfaces” based on immobilized nanoreactors that produce antibiotics “on demand.” This strategy is very promising as they are only active when needed and can be designed to be sensitive and responsive to specific stimuli, such as enzymes[[qv: 11b]] or external substrates.[[qv: 14a]]

### 2.5 Avoidance of Bacterial Resistance

Development of bacterial resistance to antimicrobial surfaces can become a major issue as it significantly limits the scope. Thus, different means are exploited to reduce the possibility that microbes become resistant to the particular effects used against them. It is a critical point that inhibition of organisms in a complex biofilm requires up to a 1000 times the antibiotic dose needed to kill bacteria in suspension.^49^ In addition, there is serious concern that the use of antibiotic‐eluting coatings released at sub‐MIC levels might promote the selection of resistant strains and result in the development of bacterial resistance.[[qv: 5b]] AMPs are a promising alternative to conventional antibiotics, because they possess broad antibiotic effects, but supposedly induce less resistance than antibiotics.[[qv: 42b]] Generally, to avoid bacterial resistance is it very important to have highly specific targets, which act on the particular bacterium that is causing the disease rather than using a wide spectrum of antibiotics.

### 2.6 Location‐Dependent Design Considerations

Depending on their locations, medical devices can be classified as: i) totally external, ii) percutaneous and permucosal, or iii) totally internal implanted devices. Totally external devices as for example contact lenses usually do not present serious risks of infection to the patient because they can either be designed for single use or allow sterilization during the utilization if necessary. Furthermore, surfaces of contact lenses are designed to provide limited adhesion of corneal endothelial cells to intraocular lenses and thus avoid cytotoxicity.^17^ Percutaneous and permucosal devices (e.g., dental implants, central venous catheters) are invasive, being partially internal to the body tissues, and therefore with high risks of infections. Ideal surfaces of percutaneous and permucosal devices have to support osseointegration and perimucosal sealing, because it is important to resist periimplant infections, for example, periimplantitis.^27^ Totally internal implant devices are usually contaminated because of specific reasons in restricted circumstances, for example, implant surface contamination before or during surgery, or hematogenous seeding from a distant infected site.^17^ Short‐term totally internal implant devices might not require a permanent coating and they can be used together with release of antimicrobials into the surrounding tissue. For long‐term totally internal implant devices (e.g., heart valves or joint replacements), stable coatings such as immobilized polymers with antimicrobial or antiadhesive properties (e.g., polysaccharides,^50^ AMPs^51^), or crosslinked polymer hydrogels^52^ protect against DAI by not dissipating over time.

## 3 Nanoscience‐Based Strategies with Antimicrobial Properties

**Figure 3:**
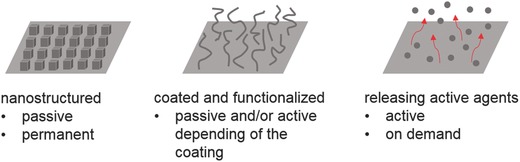
Nanoscience‐based strategies can be used to create various surfaces to protect passively or actively against bacteria colonization and proliferation.

Antimicrobial effects of surfaces can be evoked chemically, either the bulk material itself or the antimicrobial compounds embedded in it^53^ and by the surface architecture and topography.[[qv: 13b,54]] Different nanoscience‐based strategies have been developed to equip surfaces with antimicrobial or bacteria repelling activity: micro‐ and nanostructured surfaces, dynamic surfaces, coated surfaces, and surfaces that release active agents (**Figure** **3**).

### 3.1 Micro‐ and Nanostructured Surfaces Inspired by Nature

**Figure 4:**
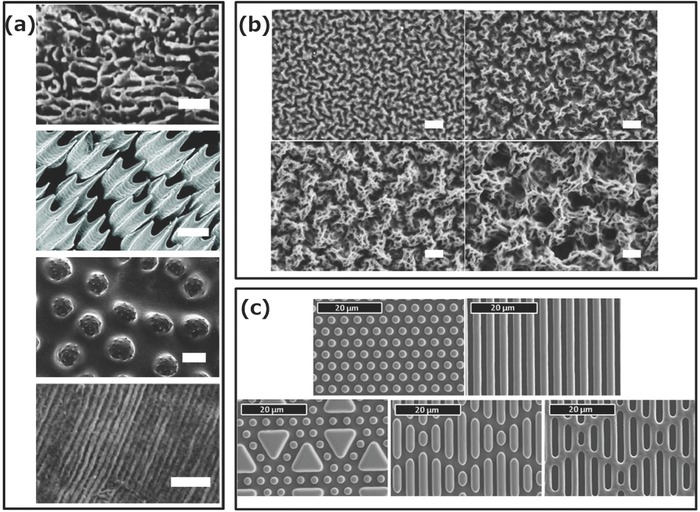
a) Surface topography of various natural models that resist fouling:^60^ pilot whale,^57^ shark,^56^ sea stars,^58^ and mussels^59^ (from top to bottom; the scale bars are 1, 100, 100, and 10 µm, respectively). b) Scanning electron microscopy (SEM) images of polyacrylic acid–polyethylene imine multilayers inspired from pilot whale skins. Scale bars: 1 µm.^64^ c) SEM images of shark skin inspired surfaces with engineered patterns on PDMS elastomers with a spacing of 2 µm.^65^ Reproduced with permission^60^ and reproduced with permission,^64^ Wiley. Reproduced with permission,^57^ Springer Nature. Republished with permission of The Company of Biologists Ltd from Wen et al.,^56^ permission conveyed through Copyright Clearance Center, Inc. Reproduced with permission,^58^, ^65^ and^59^ Taylor & Francis.

Micro‐ and nanostructured surfaces that hinder bacterial adhesion but do not kill bacteria are also found in nature. Surface microstructures represent passive mechanisms, which are nontoxic since no biocides or inhibiting agents are released to the environment. Various organisms utilize such strategies for defense against bacterial colonization, and these have inspired the development of biomimetic antibacterial surfaces.^55^ Models of surface textures from sea organisms, such as sharks,^56^ pilot whales,^57^ sea stars,^58^ and mussels,^59^ have been investigated because these animals have few problems with fouling organisms. The skins of these different animals are patterned with special microstructures (**Figure** **4**a),^60^ and the spacing between them is regarded as a key property for inducing antifouling performance.^61^ The ridged platelet structures on shark skin,^56^ for example, are considered to be a key factor in the prevention of biofouling^62^ and hierarchically wrinkled surfaces remained free of fouling for more than a year in field tests.^63^

These architectures are mimicked by engineering surface structure and hydrophobicities similar to the examples found in nature. The first step in this bioinspired strategy was to obtain micropatterns on surfaces that mimic the microstructured surfaces of the natural examples already explored. However, this strategy can be applied at the nanoscale level whether by nanopatterns or when nanometer‐size assemblies (particles, micelles, vesicles, and tubes) are combined with the micropatterns. Therefore, we include here the micropattern architectures as an inspiring and essential step in obtaining antimicrobial surfaces based on the passive strategy. Various patterns, such as pits, pillars, ribs, channels, and ridges have been produced using photolithography (Figure 4c)^62^ with a constant spacing of 2–20 µm between organized nanosized features.^62^, ^65^ Attachment of *Cobetia marina*, a microorganism larger than these surface features, was two orders of magnitude lower on the structured surface than on smooth polydimethylsiloxane (PDMS).^65^ Sharklet, a product inspired by shark skin, is already being marketed and has been shown to reduce settled microorganism density by 86% compared to a smooth surface.^62^

The textures of pilot whale skin have also been tested for their nonfouling properties,^57^ and nano‐ and microstructure coatings that mimic whale skin have been fabricated by multilayers of spray‐coated polyacrylic acid and polyethyleneimine (Figure 4b).^64^ Studies on the relationship between feature size and antifouling property indicated that the lowest level of attachment was for structures of the order of 2 µm (similar to the feature size of the skin of pilot whales and smaller than zoospores).^64^ Another example of bioinspired synthetic surface pattern was obtained by replicating the structure of macroalgae. A macroalgae mold in PDMS was filled with epoxy doped with furanone (C_4_H_4_O_2_) to obtain artificial microstructured surfaces, which showed 40% less biofouling than pure epoxy blanks, and thus demonstrated that both the chemistry and topography affect antifouling properties.^66^ Using microwave plasma chemical vapor deposition on a silicon surface, diamond nanocones have been engineered to mimic the topography of cicada fly wings. Although this structure did not inhibit the development of bacteria, it killed up to 18% of them at the surface.[[qv: 15a]] Novel approaches served to develop nanostructured surfaces with antimicrobial properties, as for example by multifunctionalization of solid supports. Poly(N‐isopropylacrylamide) (PNIPAAm) brushes were nanopatterned and allowed spatial attachment of biocidal quaternary ammonium salt molecules in the intervals between the polymer brushes,^67^ and silicon nanopillars were functionalized with quaternized polymer brushes.^68^ Such hybrid surfaces represent model systems with high potential because their ability to undergo noncovalent, dynamic, and reversible structural changes serving to control the bioactivity against bacteria in response to changes in temperature. This concept has been further developed by fabrication of PNIPAAm/lysozyme hybrid surfaces, which exhibited biocidal and fouling release properties.^69^ Such nanopatterned PNIPAAm brushes, produced by interferometric lithography followed by surface‐initiated polymerization, allowed adsorption of lysozyme into the polymer‐free regions of the substrate between the brushes: a multifunctional hybrid surface with switchable antimicrobial activity and fouling‐release ability in response to the change of temperature has been obtained. This strategy has also been combined with nanostructuring by functionalizing silicon nanopillars with brushes that release lysozyme on demand.^70^ Both micro‐ and nanopatterning of surfaces and their smart combination with antibacterial compounds have high potential after further optimization to provide more advanced solutions for the diversity of bacterial strains.

### 3.2 Microbe–Surface Interactions

An understanding of the interactions between bacteria and micro‐/nanostructures can help the development of more effective designs for antibacterial surfaces. Exploration of the factors that elicit antibiofouling properties revealed relationships between surface microstructures and bacteria colonization.^71^ Several mechanisms, such as contact area reduction (attachment point theory)^72^ and hydrodynamic variations,^73^ have been proposed as being responsible for controlling interactions between bacteria and hosts. For example, static bioassays of different sized algal species (*Fallacia carpentariae, Nitzschia* cf*. paleacea, Amphora spp*., and *Navicula jeffreyi* with dimensions from 1 to 14 µm) on polyimide surfaces with 2 or 4 µm ripples or 4 µm peaks showed the lowest level of attachment on microstructures that were slightly smaller than the algae,^72^ which agrees with reports on the effects of changing the feature size in the surface microstructures.^62^, ^65^ Different levels of interactions between surface patterns and bacteria have been identified according to bacteria size and microstructure. While these interactions have been studied by using microstructured patterns on surfaces, they indicate general properties that should be implemented at nanoscale as well, as for example generation of surfaces with a reduced contact area. Such surfaces can integrate a micropattern on which are attached nanoassemblies, such that they further decrease the point‐to‐point contact area or induce a change in the local charge expected to decrease the interaction with the bacteria.

In order to gain a better understanding of the interactions between these parameters, the physical properties of microstructured surfaces have been further divided into subgroups, such as size, shape, spacing distance, and organization of the microstructures.^74^ However, it becomes increasingly clear that the effect of surface topography on bacterial attachment is the result of an interplay of several parameters,^74^ including surface chemistry, charge^75^ wettability, and the individual morphology of the bacteria.[[qv: 15b]] In this respect, association of micro‐ and nanostructures on the same surface, a more detailed control of molecular parameters, is expected to allow a fine tuning of the properties and therefore of the bacterial interactions. This complexity might also be the explanation for some of the controversial conclusions that have been published. The lack of reports comparing the effect of such patterned surfaces on different strains makes even more difficult an overview of the trends associated with the passive strategy. Nevertheless, surface roughness and microstructures play an important role in deterring bacterial attachment, although the links between the physical factors of the surface topography and bacterial attachment require further investigation. A deeper insight at nanometer scale of such microstructured surfaces and the association of micro‐ and nanopatterns represent key aspects to be explored for the improvement of the efficacy of the antimicrobial surfaces.

### 3.3 Synthetic Micro‐ and Nanostructured Surfaces

**Figure 5:**
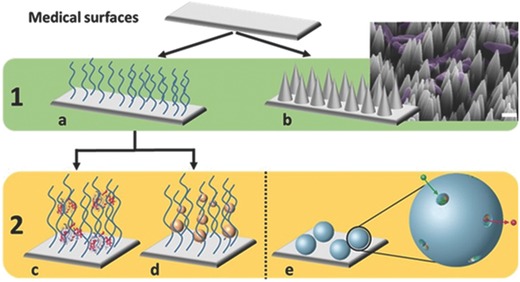
A summary of possible synthetic surface nanofunctionalization approaches for preventing DAI. 1) Antifouling properties: surfaces can be a) coated with organic compounds or b) patterned (scale bar = 1 µm)[[qv: 15a]] to avoid growth of bacteria. 2) Antimicrobial properties are provided via c) peptides/drugs or d) nanoparticles entrapped in the organic coatings. Another antimicrobial solution could be e) the direct grafting of a nanoreactor onto the surface to control drug release.[[qv: 14a]] Adapted with permission.[[qv: 15a]] Copyright 2016, American Vacuum Society. Adapted with permission.[[qv: 14a]] The Royal Society of Chemistry.

Another direction in fighting biofilm formation is based on synthetic approaches for inducing a direct change in the surface structure of the device (**Figure** **5**b) [[qv: 14a,15a]] via material science or mechanical methods. This approach to inhibit bacterial growth is based on the concept of rough surfaces without directly mimicking natural patterns.^76^ They are less advanced than the coating procedures described in Section 3.5; nevertheless, their potential is increasing as surface modification is a promising and growing research field. Nanostructured surfaces for instance can be created via direct addition of nanoobjects to kill bacteria once they reach the surface. High‐aspect ratio surfaces have been produced with silicon nanopillars patterned by the deep reactive ion etching technique with SF_6_ and O_2_ gases in the etch cycle and C_4_F_6_ gas in the deposition cycle. These silicon nanopillars, with random interspaces, increased the contact angle of a silicon surface from 75° to 154° and led to up to 86% death of bacteria on their surfaces.[[qv: 13b]] Moreover, the antibacterial activity of silicon nanopillars can be further enhanced by decoration with silver or copper nanoparticles^77^ or quaternized polymer brushes.^68^ Creation of micro‐ or/and nanostructured roughness can decrease bacterial growth by generating very hydrophobic surfaces.^24^ The topography can be shaped with biocompatible polymers, such as PDMS, polystyrene, polycarbonate, or polyethylene to obtain a desirable roughness and hydrophobicity with contact angles increasing from 60° to 90° before to 150° after restructuring.^78^ These structured surfaces massively decrease bacteria retention to <0.1% compared to the initial concentration, while unstructured surfaces retained 34%. Surfaces have also been patterned with different microscale motifs (pillars, cross pillars, hexagonal pillars, and hexagonal pits) to inhibit bacterial growth to 11% of coverage compared to the control surface.^48^ Capture and killing of bacteria has been achieved by functionalization of silicon nanopillars with bacteria‐binding molecules^79^ and functionalization with polymer brushes entrapping an antibacterial enzyme, lysozyme.^70^ The topographical approach to preventing biofilm formation by i) controlling surface roughness and ‐pattern to prevent bacteria adherence, ii) adding patterned nanopillars to kill bacteria, or iii) to further functionalize the nanopatterned architecture with actively antimicrobial moieties shows promising results in antifouling and bactericidal properties, and thus represents a solution with high potential for protecting medical devices from pathogen infections.

### 3.4 Dynamic Surfaces with Antifouling Properties

Whereas the creation of nanostructures represents a static approach to the production of antibacterial surfaces, living organisms also fight bacterial attachment by using approaches based on induced dynamic surface deformation or active motion.^80^ Such dynamic strategies have been developed by many marine organisms, including mollusks and corals to combat the attachment of fouling from algae, slime, and encrusting organisms. For example, the surfaces of mollusks and coral are protected against fouling organisms by motion of hair‐like cilia.^81^ These act as barriers against invading particles, since dynamic deformation and cilia provide unstable surfaces that are unfavorable for bacterial attachment. Based on this concept, researchers have used various external stimuli, including electrical, mechanical, and pneumatic, to induce dynamic surface deformations that are able to detach both biofilms and macrofouling organisms.^82^ Investigations of the effectiveness of dynamic surfaces driven by pneumatic actuation against marine biofouling have demonstrated biofilm detachment (>90%) in both laboratory and field environments.^83^ Recently, this approach has been applied to implant devices by introducing two inflation lumens into the tube walls of the silicone elastomer of a urinary catheter.^84^ The presence of external stimuli, such as hydraulic or pneumatic actuation, results in repeated deformation of the inner surface of the catheter and the active removal of >80% infectious biofilms.^85^ Such dynamic surfaces are active after biofilm formation or bacterial attachment rather than by directly killing the cells or inhibiting bacterial growth. Therefore, it is necessary to supplement this strategy with other antibacterial treatments, since accumulation of dead bacteria on the surface provides nutrition for subsequent infection in the long‐term use of implant devices. Furthermore, dynamic surfaces usually require external stimuli to drive the surface deformation or motion, and more practical technologies for producing such stimuli still need to be developed.

### 3.5 Coated Surfaces with Antibacterial Properties

Alternative synthetic strategies to obtain antiadhesive and antimicrobial surfaces are based on: coating surfaces with i) organic compounds and ii) inorganic metals. (Figure 5).

#### 3.5.1 Polymer‐Coated Surfaces

Surfaces coated with organic compounds are based on: i) using the properties of the compounds themselves to modify the surface or ii) attachment of active antimicrobial compounds on the surface.

The major synthetic approach for preventing biofilm formation is the modification of surfaces with organic compounds, often biocompatible polymers,^86^ which directly reduce bacteria adhesion to the device (Figure 5a). For example, polyamide reverse osmosis membranes are protected from the attachment of bacteria through a phosphorylcholine block copolymer coating.^87^ This is sufficient to reduce bacterial growth by at least a factor of 10. Antifouling strategies^88^ have also been developed with polymer brushes^89^ and with amphiphilic block copolymer coatings, such as poly(styrene)‐*block*‐poly(ethylene‐*ran*‐butylene)‐*block*‐poly(isoprene) with polyethylene glycol (PEG)‐hydrocarbon sidechains which induce a tenfold decrease in microorganism density on their surfaces compared to surfaces without amphiphilic block copolymer coatings.^53^ Moreover, nanopatterned polymer brushes were exploited as they allow antibacterial properties to be triggered by external stimuli if the copolymers are appropriately selected to be stimuli responsive. This concept has been implemented by using thermoresponsive poly(*N*‐isopropylacrylamine) brushes that expose patterns of biocides^67^ or antimicrobial enzymes.^69^ In addition, it was also demonstrated that thermoresponsive, nanopatterned polymer brushes allow triggered removal of debris after effective killing of bacteria.^90^ Moreover, chemical modification with polymers has also been realized on surfaces that were previously patterned with silicon nanowires, further enhancing the antibacterial properties.^70^, ^79^ Besides synthetic polymers, antifouling properties have also been obtained with polysaccharide coatings that decreased bacteria adhesion to a titanium surface up to 96% after 90 min of contact,^50^ and agarose crosslinked on a solid support reduced the adhesion of proteins to device surfaces by >90%.^91^ Triggered antimicrobial activity was also achieved with carbohydrate‐based compounds that switch antibacterial activity upon illumination with UV–vis light.^92^

The physical property of the coating compound is sometimes coupled to antimicrobial effects from the chemical nature of the polymer; for example, a mixture of hyaluronic acid and chitosan^93^ or poly(*N*‐hydroxyethylacrylamide) crosslinked with salicylate^94^ decreased bacteria adhesion, and also inhibited their proliferation. It is important to engineer surfaces that are able to prevent bacteria adhesion and protein absorption at the same time in order to successfully impede biofilm formation. Furthermore, a decrease in several orders of magnitude of bacteria attachment is needed in order to prevent biofilm formation over longer period of times, as bacteria are proliferating exponentially with short generation times.

#### 3.5.2 Surfaces‐Releasing Active Agents

Another way of preventing biofilm formation is to actively kill bacteria in close proximity of the surface. This can be done by releasing active agents like small molecules or ions into environment around the surface.

*Organic Antimicrobial Agents*: Most developed solutions for coatings against DAI are based on layers that entrap antimicrobial agents^18^, ^95^ to provide controlled release of drugs (Figure 5c). Antibiotics directly incorporated in polymer coatings resulted in a controlled and constant release of the drug during 7 d and induced a decrease in DAI in animal models.^96^ However, with these strategies it has to be taken into account that depending on the release properties, the concentration of released antibiotics will eventually drop below the MIC. Peptides immobilized on polydopamine coatings have been used to prevent bacteria adhesion.^97^ Alternatively, peptides were entrapped in polymer matrix coatings on surfaces to increase the antimicrobial effect of free peptides and to keep them at the interface of devices with their biological environment for up to one month. This controlled their release without producing possible toxicity to mammalian cells.^98^ Using compounds that are degraded by pathogens to release drugs to inactivate bacterial growth is another very interesting approach,^[11b]^ for example, the destruction of polysaccharide multilayer films with entrapped AMPs by secretions of bacteria and yeast led to peptide release and resulted in the destruction of the pathogens.^32^

A recent development has been the use of drug encapsulated vesicles^99^ or surface immobilized nanoreactors.[[qv: 14a]] Micro‐ or nanospheres of polylactic acid–polyvinyl alcohol were loaded with usnic acid, the release of which prevented biofilm formation by reducing the amount of bacteria by >10,000 times after 72 h incubation with *S. aureus*.^23^ Our group has developed self‐defending surfaces that locally produce antibiotics “on demand” and at a controlled rate based on immobilized nanoreactors (Figure 5e).^14^ Here, enzymes were encapsulated inside polymer vesicles and transport of substrates/antibiotics through the membrane was facilitated by insertion of pore proteins. These active surfaces produce and release of antibiotics that inhibit bacterial growth in their surroundings for up to 7 d by adding the required amounts of substrate to the outer medium.

*Inorganic Antimicrobial Release*: Biofilm formation can also be prevented by directly using the properties of metallic elements, such as zinc,^100^ selenium,^101^ copper, or silver,^102^ incorporated in or grafted on the surface of medical devices (Figure 5d). The use of silver ions in their most common oxidation state (Ag^+^)^103^ is the predominant inorganic approach that has been developed^46^ with rare induced microbial resistance, only known in Gram‐negative bacteria.^104^ However, the complex antimicrobial mechanism of silver nanoparticles (AgNPs) is not yet fully understood.^105^ AgNPs can be synthesized in an eco‐friendly way^106^ and are nowadays the main inorganic nanoscience‐based surface modification of medical devices.^10^, ^107^ These nanoparticles have the advantage of being a stable reservoir of antimicrobial silver ions and thus having longer use than classical free Ag^+^ ions.[[qv: 9a,43]] AgNPs are usually functionalized with chemical agents to improve their aqueous stability and dispersion[[qv: 4b,9a,108]] before being linked to device surfaces that have been prefunctionalized with polymers,^10^ hydrogels,[[qv: 9a]] chitosan,[[qv: 4b]] or silicon nanowires.^77^ These strategies allow entrapment of AgNPs at the interface between the medical device and the biological environment and release Ag^+^, the active bactericide state of silver.^43^ AgNPs have shown very high antimicrobial properties by decreasing the bacteria present on chitosan gel[[qv: 4b]] or polymer brushes^10^ by >99.8% 24 h after exposure. Ag^+^ release was demonstrated to be an important parameter in long‐term antibiofilm activity, reducing bacteria adhesion and proliferation in vitro,[[qv: 4b]] and even leading to an in vivo decrease of DAI in rats^43^ as well as inhibiting the toxic effect of free silver ions by storing them in the zero oxidation state (Ag^0^) as AgNP.[[qv: 9a]] It was demonstrated that AgNPs have slower in vitro antimicrobial activity (at the same total silver concentration: 10 mg L^−1^) than free silver ions, because of the release kinetics of Ag^+^ from the AgNPs.^108^ Even though AgNPs have not shown any direct toxicity thus far, mainly because of prior coating of the NPs with chemical agents or of the biocompatible surfaces in which they were entrapped,[[qv: 9a,43,109]] they are discussed controversially regarding platelet aggregation in vivo. Furthermore, they are reported to possibly cause hemolysis, mitochondrial perturbation, or increase oxidative stress which possibly induces cytotoxicity.^110^

Some other solutions are emerging from inorganic nanoscience. Thin‐film assemblies of silica and magnetite nanoparticles with salicylic acid and antibiotics showed a 10^3^‐fold decrease in bacteria development.^111^ Also, carbon nanotubes containing the cell wall degrading enzyme lysostaphin killed 99.9% of bacteria after 2 h of exposure without releasing the enzyme trapped in the nanotubes.^112^ However, further experiments on such solutions are necessary both to improve their antibacterial activity and to evaluate the effects of long‐term exposure in the case of carbon nanotubes. An additional promising nanostrategy that is being developed is to not only protect, but also repair damage; for example, combining the antimicrobial properties of Ag with calcium phosphate nanoparticles to remineralize tooth damage.^113^ There is no doubt that in the coming years inorganic nanosolutions for preventing biofilm formation will continue to be developed, although it will also be necessary to assess the potential long‐term cytotoxicity of such nanomaterials.

## 4 Conclusion

In nature, surfaces that impede biofilm formation based on special compositions or topography have been discovered and inspired the development of synthetic antimicrobial surfaces with the tools of nanoscience. Active (bacteria killing) or passive (preventing bacteria attachment) strategies provide new solutions to effectively reduce DAI. Owing to the advances in nanoscience, smart surfaces that act in a special and time‐restricted area, and therefore able to lower doses and side effects were introduced. Moreover, an improved understanding of surface–microbe interactions at the micro‐ and nanoscale allows to engineer surfaces without any active agents needed, thereby eliminating side effects. In addition to conventional antibiotics, AMPs were recently introduced as an elegant approach that avoids both toxicity and bacterial resistance. However, there is still a long way to go in the development of effective, ecological, and economic antimicrobial surface strategies. Various antimicrobial surfaces with specific topography have been fabricated to obtain efficient and long‐term antifouling properties. Micro‐ and nanopatterns represent a passive approach and are generally less toxic than antibiotic‐releasing surfaces. However, most of the current fabrication methods for producing such patterns are still too complex and involve high costs. In addition, the domain of antimicrobial surfaces is still controversial due to the biocomplexity of the medical conditions and a lack of standardization of the characterization methods and functionality of such surfaces. Therefore, we limited our review to examples that support the first step of surface modification and indicated the advantages and limitations still to be solved but without details related to a specific application. A multitude of different medical applications for antibacterial and antimicrobial surfaces is evident; however, more recently new application areas have emerged, such as antimicrobial semiconductors on textile surfaces.^114^ It is clear that the biospecificity of the application is inducing supplementary requirements the functionalized surface should cope with, but they are not the focus of this review.

Compared to conventional surface coatings, there is at present no easily applicable solution for the production of surface patterns, and the necessary studies of the in vivo impact of patterned medical devices have yet to be performed. Furthermore, it is necessary to gain specific understanding of the interaction between different types of surfaces and their antifouling and contact killing properties. Depending on where the device is to be implanted, specific tissue effects have to be considered. The balance between benefits versus drawbacks and potency versus toxicity of antimicrobial strategies needs to be evaluated, as dependence on a single strategy is not adequate for fighting the formation of biofilms on implanted devices. Furthermore, in vitro studies alone are not sufficiently reliable for evaluating the in vivo performance of antimicrobial surfaces. Due to numerous different requirements, it is not surprising that the importance of various factors such as the influence of surface roughness or structure is still controversial. Very few studies have focused on the long‐term toxicity of antimicrobial coatings or on potential pathogen resistance. On one hand, a low concentration of antimicrobial substrates is preferred in order to minimize toxicity, whereas on the other hand, sublethal doses of antibiotics will enhance biofilm formation. Moreover, in the case of inorganic nanoparticles, long‐term degradation might induce unwanted side effects. While, AgNPs coated on surfaces induce less toxicity than free‐floating AgNPs, there have been no reported investigations of the possible detachment of AgNPs from coated medical devices, even though such nanoparticles show toxicity in high concentrations. Thus, it is crucial to study not only the possible toxicity of coatings, but also their detachment, biodistribution, and degradation. The future for biofilm control on surfaces is expected to be performed by novel nanoscience‐based strategies that address the surface structure to inhibit attachment or induce contact killing, as well as by “smart surfaces” which are able to locally fight bacterial attack “on demand.” Integrated and multiple defense mechanisms must be considered and used when designing antibacterial coatings for implant devices. Several passive or active strategies have been developed, but only a few multifunctional approaches incorporate both of these approaches. Finding and evaluating optimal antibiofilm surfaces require a multidisciplinary approach supported by industrial partners, material‐, and healthcare‐scientists. Nevertheless, nanoscience‐based solutions against DAI are expected to cope at a molecular level with the complex processes involved in biofilm formation. Overall, more studies in clinical settings, ultimately including those with a clinical outcome, are required since efficacy strongly depends on the type and length (short or long term) of the clinical application.

## Conflict of Interest

The authors declare no conflict of interest.
